# Fourteen-year follow-up of a child with acroscyphodysplasia with emphasis on the need for multidisciplinary management: a case report

**DOI:** 10.1186/s12881-020-01127-6

**Published:** 2020-09-29

**Authors:** Katina Kartalias, Austin P. Gillies, Maria T. Peña, Andrea Estrada, Dorothy I. Bulas, Carlos R. Ferreira, Laura L. Tosi

**Affiliations:** 1 The George Washington University School of Medicine and Health Sciences, Washington, DC, USA; 2 Bone Health Program, Division of Orthopaedics & Sports Medicine, Children’s National Hospital, 111 Michigan Ave. NW, Washington, DC 20010 USA; 3 Division of Otolaryngology, Children’s National Hospital, Washington, DC, USA; 4 Division of Endocrinology and Diabetes, Children’s National Hospital, Washington, DC, USA; 5 Department of Radiology, Children’s National Hospital, Washington, DC, USA; 6 Skeletal Genomics Unit, National Human Genome Research Institute, National Institutes of Health, Bethesda, USA

**Keywords:** Acroscyphodysplasia, Skeletal dysplasia, Cone-shaped epiphysis, Multidisciplinary management, Rare disorder

## Abstract

**Background:**

Acroscyphodysplasia has been described as a phenotypic variant of acrodysostosis type 2 and pseudohypoparathyroidism. In acrodysostosis, skeletal features can include brachydactyly, facial hypoplasia, cone-shaped epiphyses, short stature, and advanced bone age. To date, reports on this disorder have focused on phenotypic findings, endocrine changes, and genetic variation. We present a 14-year overview of a patient, from birth to skeletal maturity, with acroscyphodysplasia, noting the significant orthopaedic challenges and the need for a multidisciplinary team, including specialists in genetics, orthopaedics, endocrinology, and otolaryngology, to optimize long-term outcomes.

**Case presentation:**

The patient presented as a newborn with dysmorphic facial features, including severe midface hypoplasia, malar flattening, nasal stenosis, and feeding difficulties. Radiologic findings were initially subtle, and a skeletal survey performed at age 7 months was initially considered normal. Genetic evaluation revealed a variant in *PDE4D* and subsequent pseudohypoparathyroidism. The patient presented to the department of orthopaedics, at age 2 years 9 months with a leg length discrepancy, right knee contracture, and severely crouched gait. Radiographs demonstrated cone-shaped epiphyses of the right distal femur and proximal tibia, but no evidence of growth plate changes in the left leg. The child developed early posterior epiphyseal arrest on the right side and required multiple surgical interventions to achieve neutral extension. Her left distal femur developed late posterior physeal arrest and secondary contracture without evidence of schypho deformity, which improved with anterior screw epiphysiodesis. The child required numerous orthopaedic surgical interventions to achieve full knee extension bilaterally. At age 13 years 11 months, she was an independent ambulator with erect posture. The child underwent numerous otolaryngology procedures and will require significant ongoing care. She has moderate intellectual disability.

**Discussion and conclusions:**

Key challenges in the management of this case included the subtle changes on initial skeletal survey and the marked asymmetry of her deformity. While cone-shaped epiphyses are a hallmark of acrodysostosis, posterior tethering/growth arrest of the posterior distal femur has not been previously reported. Correction of the secondary knee contracture was essential to improve ambulation. Children with acroscyphodysplasia require a multidisciplinary approach, including radiology, genetics, orthopaedics, otolaryngology, and endocrinology specialties.

## Background

Acroscyphodysplasia, a unique form of metaphyseal dysplasia, has been described as a phenotypic variation of pseudohypoparathyroidism and acrodysostosis type 2 [1]. First reported in 1968, acrodysostosis is a group of rare autosomal dominant genetic disorders [2] that present with skeletal, endocrine, and neurological features encompassing a broad range of skeletal dysplasias [3]. The skeletal features may include brachydactyly, facial hypoplasia, cone-shaped epiphyses, short stature, and advanced bone age [3], which characterize a spectrum of disorders, including Albright’s Hereditary Osteodystrophy (AHO), pseudohypoparathyroidism (PHP) type 1, pseudopseudohypoparathyroidism (PPHP), and metaphyseal acroscyphodysplasia [4, 5]. Facial hypoplasia, if severe, can contribute to or result in severe obstructive sleep apnea (OSA). The endocrine manifestations may include resistance to multiple hormones, notably parathyroid hormone (PTH) or thyrotropin [3]. Neurologically, the affected individuals may demonstrate mild-to-moderate intellectual disability [3].

Acroscyphodysplasia is distinguished by long bone epiphyses embedded in a schypho- (‘cup’ in Greek) shaped metaphysis, seen in conjunction with short stature and brachydactyly [1]. The Nosology and Classification of Genetic Skeletal Disorders: 2019 revision classifies acrodysostosis as an acromelic disorder caused by genetic variations in *PDE4D* and *PRKAR1A* [6]. Michot et al. emphasized the relatively frequent occurrence of a cup-shaped epiphysis phenotype [4], which is a deformity most commonly seen in the fingers, and to a lesser degree in the central aspect of the distal femurs, proximal tibias, and humeri [4].

To date, virtually all of the literature on acroscyphodysplasia focuses on phenotypic findings, endocrine changes, and genetic variation characteristic of this disorder; only a few reports discuss skeletal deformities or orthopaedic interventions [7–9]. In this report we present a 14-year overview of a female patient with acroschyphodysplasia, from birth to skeletal maturity, including genetic analysis and clinical management, with an emphasis on the orthopaedic interventions necessary to maintain independent ambulation. We report posterior tethering/growth arrest of the posterior distal femur for the first time, which appeared asynchronously and disproportionately in the right leg and led to significant challenges in the orthopaedic management. Our work emphasizes the importance of a multidisciplinary approach, involving the genetics, orthopaedics, endocrinology, otolaryngology, and radiology specialties, in the management of these patients in order to optimize the long-term outcomes.

## Case presentation

The patient was born with dysmorphic facial features, including severe midface hypoplasia, malar flattening, and nasal stenosis. Initial clinical management focused on her significant breathing and swallowing difficulties. She required a gastrostomy tube for feeding and was followed closely by the otolaryngology team.

**Fig. 1:**
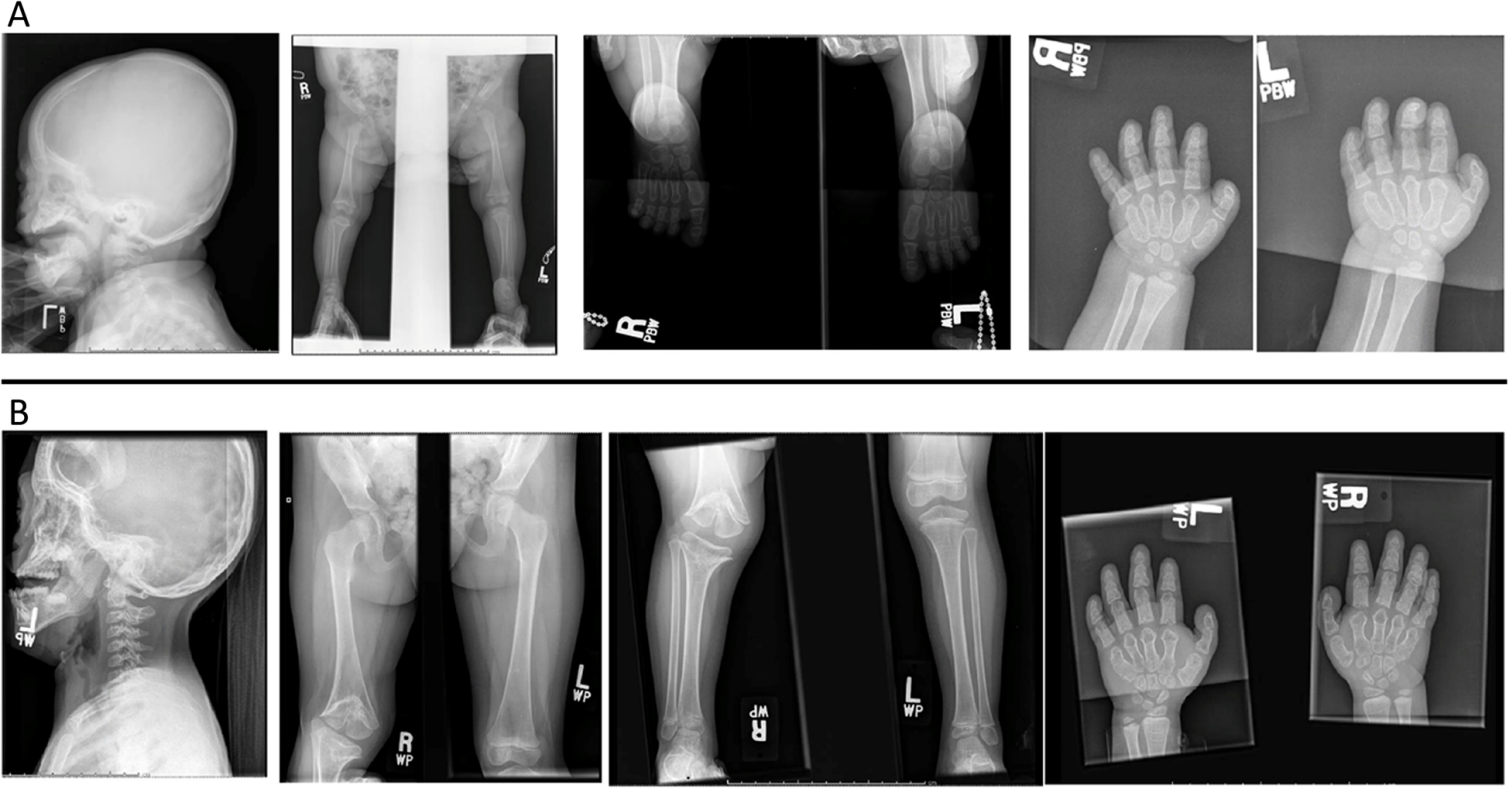
**a** The first row includes images from the skeletal survey performed at age 7 months, which show brachycephaly, mild midface hypoplasia, right distal femoral and proximal tibial coned epiphyses, brachydactyly, and advanced bone age with ossification of the carpal and tarsal bones. **b**: The second row includes images obtained at age 33 months, demonstrating the significant progression of midface hypoplasia, brachycephaly, and cone shaped epiphysis in the right knee, brachydactyly, and advanced bone age

While she was in the nursey, the neonatology team focused on understanding her midface hypoplasia and respiratory issues. MRI and CT scans of the brain were unremarkable except for simplified cerebral gyri and absence of synostosis in the major bone structures. She was seen by the genetics team at age 7 months, who concluded that her nasal cartilage hypoplasia along with rhizomelic shortening clinically supported the diagnosis of chondrodysplasia punctata. An initial skeletal survey was interpreted as a normal study with no evidence of epiphyseal stippling (Fig. 1a). (A review several years later revealed brachycephaly, mild midface hypoplasia, right distal femoral and proximal tibial cone-shaped epiphyses, brachydactyly, and advanced bone age with ossification of the carpal and tarsal bones.) Initial genetic testing included sequencing for *FGFR3* (fibroblast growth factor 3), *ARSE* (arylsulfatase E), and multiple genes associated with craniosynostosis, all of which were negative. No other genes associated with chondrodysplasia punctata were analyzed. Measurement of plasmalogen revealed normal concentrations, thus ruling out peroxisomal defects as an underlying cause. Microarray analysis at age 2 revealed an interstitial deletion of 1.2 Mb within the cytogenetic band 17q11.2 encompassing the *NF1* (neurofibromatosis 1) gene. It was noted that the child had coarse bright red hair and diffuse freckling. While there was debate whether some areas of freckling represented café-au-lait spots, there was consensus that she had fewer than 6 café-au-lait spots, no axillary or inguinal freckling, and no evidence of neurofibromas, plexiform neurofibromas, Lisch nodules, or boney lesions consistent with NF1. Her facial dysmorphism was due to a shallow facial profile, not sphenoid dysplasia and repeat brain MRI did not reveal stigmata of NF1. While she did have short stature and cognitive delay, the diagnosis of NF1 was ruled out as she did not fulfill any diagnostic criteria and the conclusion at this time was that she had an unknown skeletal dysplasia.

### Orthopaedic management

**Fig. 2:**
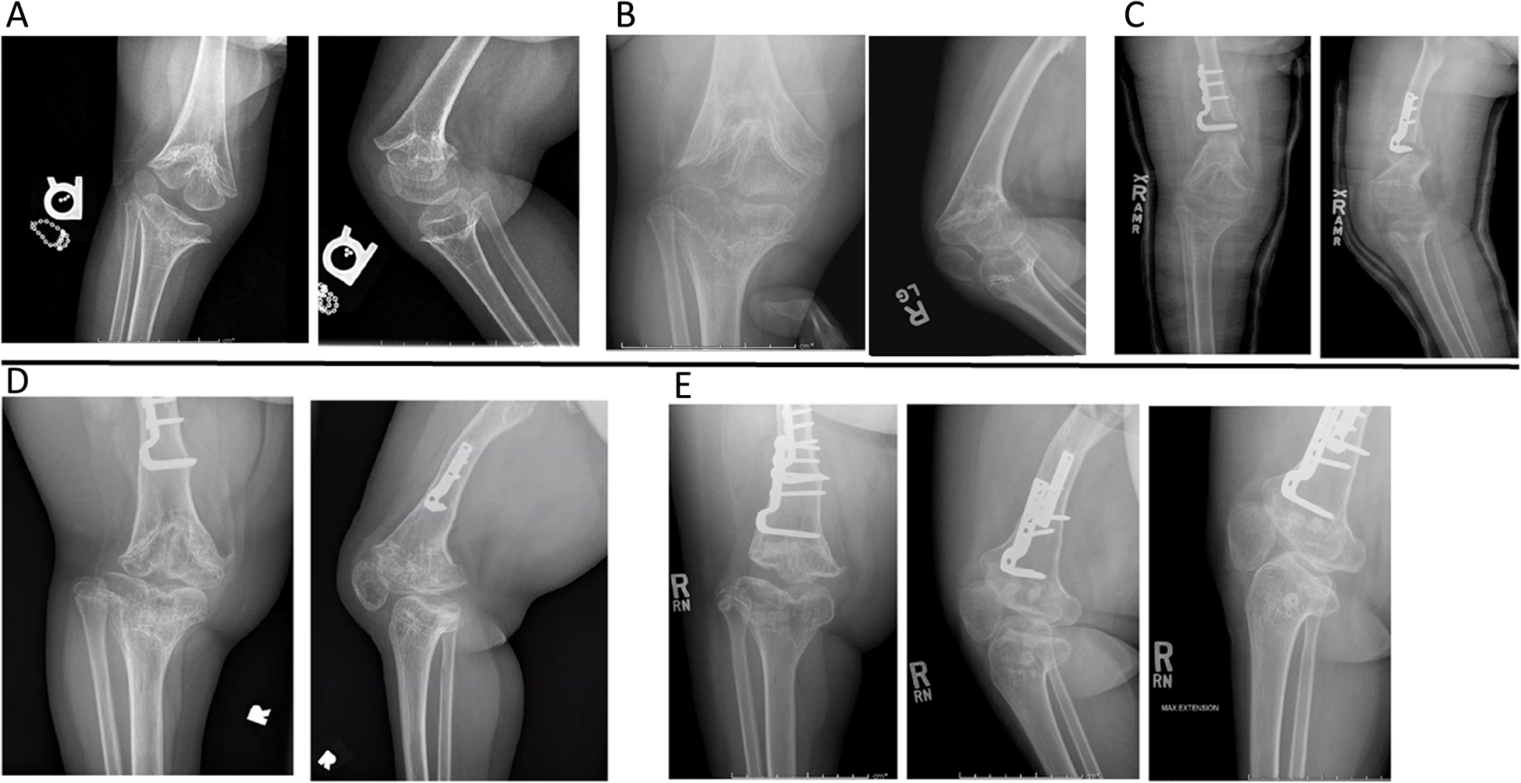
**a** Right knee films, 2 years 9 months/ **b:** Right knee films, 5 years 7 months. **c:** Right knee films, 5 years 10 months. **d:** Right knee films, 10 years 11 months. **e:** Right knee films, 13 years 10 months

The child presented to the general orthopaedic clinic at age 2 years 9 months for evaluation of a severely crouched gait, leg length discrepancy, and a right knee contracture > 45 degrees. A repeat skeletal survey was performed (Fig. 1b), including the right knee (Fig. 2a). Radiographs revealed progression of the cone-shaped epiphyses of the right distal femur and proximal tibia embedded in their respective metaphyses, with no evidence of growth plate changes on the left leg (Fig. 1b). Additionally, the right patella was laterally subluxed.

#### Right lower extremity

The child was referred to our multidisciplinary skeletal dysplasia clinic at age 3 years 5 months. The severe right knee contracture was thought to include a component of muscle contracture and, therefore, the initial orthopaedic intervention consisted of right medial and lateral hamstring lengthening and posterior capsulotomy. This was followed 3 weeks later with lateral release and medial plication to correct the patellar dislocation. Postoperatively, her contracture measured − 20 degrees, and significant improvement in her ambulation was noted.

Over the next 2.5 years, despite extensive bracing, her right knee contracture recurred to − 50 degrees and was now recognized to be primarily caused by bowing of the distal femur. Radiographs revealed that the femoral growth plate was sloping anteriorly, with tethering and premature closure posteriorly (Fig. 2b). Given her young age, she underwent a right distal femur extension/anterior closing wedge osteotomy at 5 years 10 months (Fig. 2c). The osteotomy proceeded to non-union and displacement, requiring spica casting to achieve solid healing, which significantly improved alignment, and she returned to independent ambulation with a walker.

As the patient grew taller, her right sided knee contracture recurred, severely limiting her function (Fig. 2d). At age 10 years 11 months she underwent a repeat extension osteotomy of the right distal femur; placement of the plate was difficult due to the shape of the distal femur, and the lateral collateral ligament (LCL) had to be released and reattached during closure.

Postoperatively, although the knee flexion contracture was fully corrected, it became clear that the LCL was insufficient, warranting a long leg splint to prevent the knee from falling into severe varus as she walked. At age 12 years 5 months she underwent LCL reconstruction. The tensor fascia lata below the knee joint was found to be severely deficient, and both the tibial and fibular components of the LCL were absent; these components were repaired using anterior tibialis allografts, leading to correction of her knee instability (Fig. 2e).

#### Left lower extremity

**Fig. 3:**
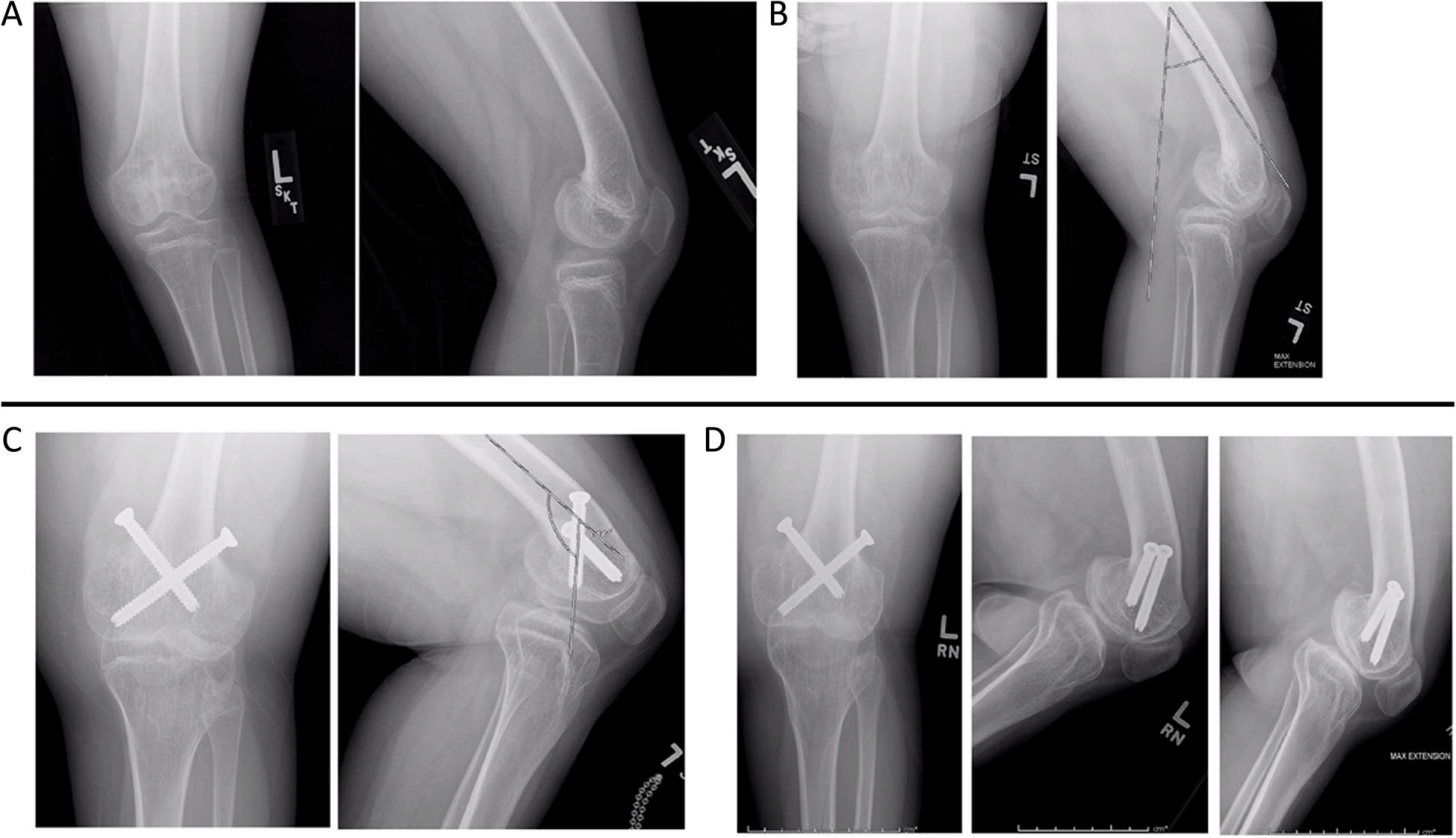
**a:** Left knee films, 2 years 9 months. **b:** Left knee films, 10 years 5 months/ **c:** Left knee films, 11 years 7 months. **d:** Left knee films, 13 years 10 months

Clinically and radiographically, the left distal femur and proximal tibial epiphyses appeared quite normal on both of the early skeletal surveys. At approximately 6 years of age, the patient began to develop a mild knee contracture, which eventually reached ~ 30 degrees (Fig. 3a). Serial radiographs did not demonstrate the cone-shaped epiphysis previously seen on the right side, but rather premature closure of the distal femoral growth plate posteriorly, with secondary bowing of the distal femur (Fig. 3b). At age 10 years 11 months, the patient underwent left distal femur anterior screw hemiepiphysiodesis in conjunction with the second extension osteotomy performed on the right side (Fig. 3c). This was done in an effort to prevent progression of her knee contracture. At age 13 years 11 months, she had full extension of the left knee clinically although radiographs indicated a slight residual contracture (Fig. 3d). As noted above, this intervention, combined with surgical correction of the right side, corrected her severely crouched gait, and she is now an independent, brace-free ambulator with erect posture. The patient’s mother was particularly pleased with the improved ambulation of her child, who no longer needed to be carried or transported in a stroller, and the nearly complete knee range of motion, which significantly improved their daily quality of life.

#### Leg length discrepancy

**Fig. 4:**
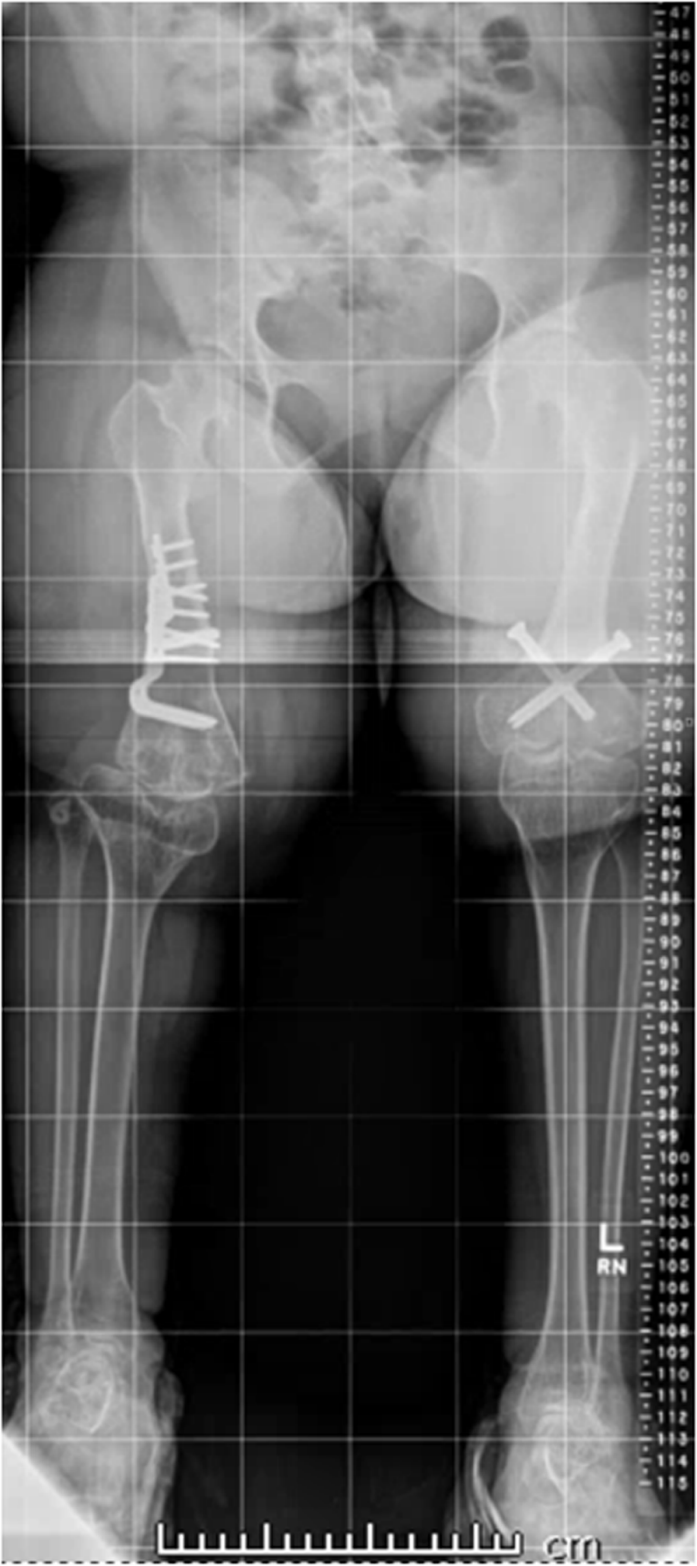
Lower extremity alignment films, at age 13 years 10 months. The image demonstrates improvement in the leg length discrepancy to just over 2 cm

The patient always showed a leg length discrepancy (Fig. 4). This has been quite variable, depending on the severity of her knee contracture. The extension osteotomies helped ameliorate this problem over time, and at skeletal maturity she requires a 2-cm lift on the right side.

#### Upper extremities

**Fig. 5:**
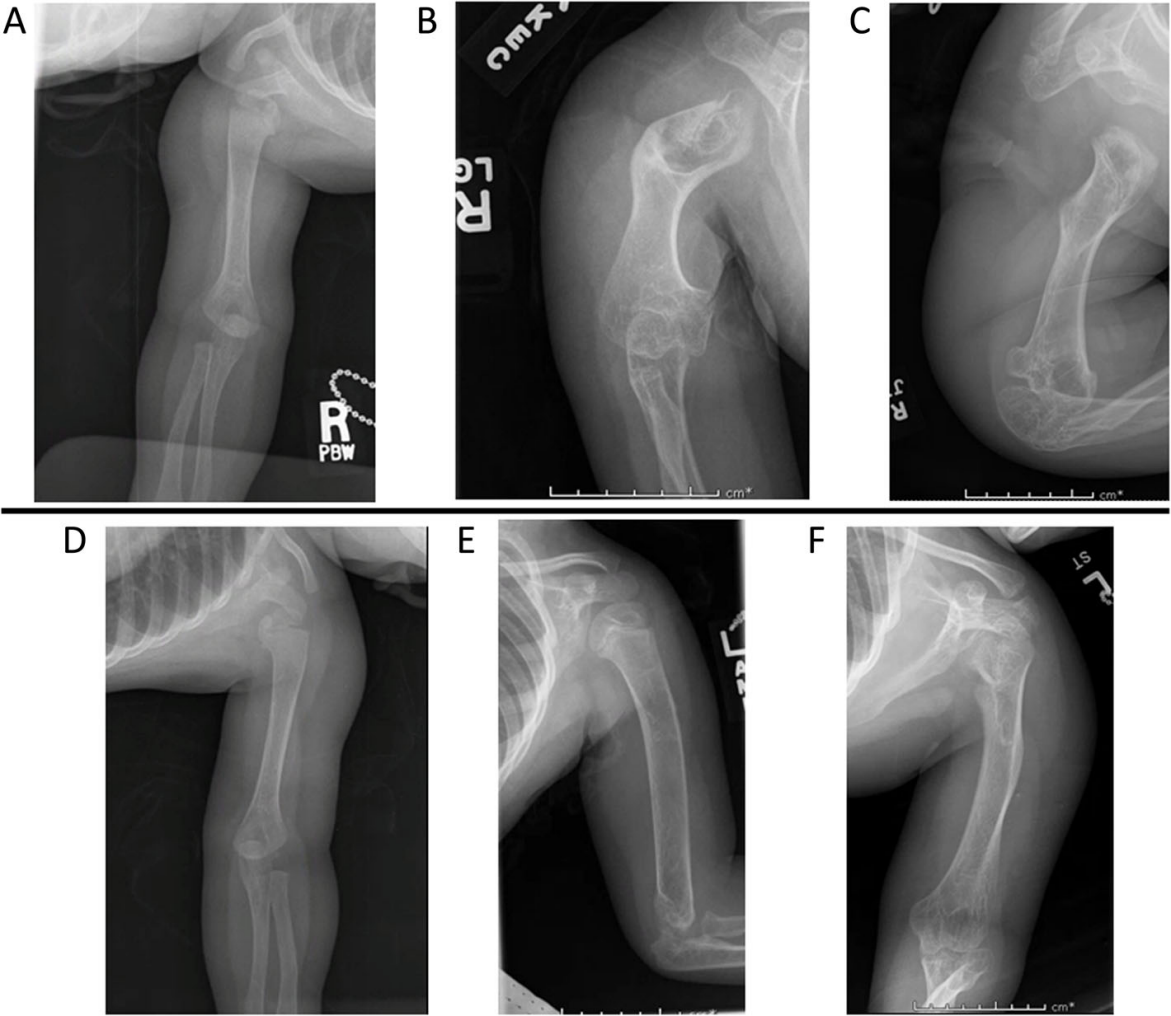
**a** Right humerus film, 7 months. **b:** Right humerus film, 4 years 7 months. **c:** Right elbow fil, 11 years 7 months. **d:** Left humerus film, 7 months. **e:** Left elbow film, 3 years 7 months. **f:** Left humerus film, 10 years 7 months

Similar to her lower extremities, the patient’s arms, particularly the humeri, showed asymmetric involvement. At 7 months of age, her humeri and forearms appeared grossly normal (Fig. 5a and d), but by 33 months the right side demonstrated abnormal proximal metaphyseal bowing (Fig. 5b and e), with broadening of the diaphysis and rhizomelic shortening; the elbow was also abnormally articulated. At maturity, clinical examination demonstrated limited range of motion of the right shoulder and lack of full extension of the right elbow (Fig. 5c and f). The patient also demonstrated brachydactyly of both the hands and feet.

#### Spine

Her spine appeared normal on the first and second skeletal survey, and at skeletal maturity she still shows no signs of spine abnormalities radiographically. Her intellectual disability limits our neurological evaluation, but no lower extremity motor weakness has been noted.

#### Bone age

Consistent with previous findings, the patient’s bone age was consistently advanced, starting with ossification of the carpal and tarsal bones noted at age 7 months. At age 12 years, she revealed complete fusion of all epiphyses.

### Genetic analysis

At age 10 years 8 months she was reassessed by the genetics team based on her more prominent clinical and radiographic findings, including the flat facial profile and nasal bridge, metaphyseal cupping, brachydactyly, and short stature, which were then felt to be consistent with metaphyseal acroschyphodysplasia and led to a confirmatory genetic diagnosis. The patient was found to carry a variant of unknown significance in the *PDE4D* gene: NC_000005.9:g.58334694A > G, NM_001104631.1:c.913 T > C, p.(Ser305Pro)(Supplementary Data Table 1) [10, 11]. Targeted molecular analysis of parents revealed that the variant was inherited de novo. This novel variant is exceedingly rare in the general population and was not found in over 126,000 individuals in gnomAD. It is extremely well-conserved through evolution, and the most common in silico algorithms predict it to be pathogenic (Polyphen-2, SIFT and CADD), with a high CADD score of 31 [12].

### Endocrine manifestations

The patient had mild PTH resistance, which was discovered once the correct genetic diagnosis was confirmed. Her initial blood investigations showed levels of intact PTH (iPTH) of 88. 9 pg/mL (13–85 pg/mL), calcium 8.9 mg/dL, and phosphorus 5 mg/dL. Her iPTH was easily normalized with calcitriol and calcium carbonate. Thyroid stimulating hormone (TSH) has since consistently been in the normal range, 3.11 μU/mL (0.51–4.91μU/mL), as well as free T4, 1.45 ng/dL (1.1–1.6 ng/dL). No other endocrine abnormalities were identified.

### Otolaryngology assessment

Due to the child’s severe midface hypoplasia, she had difficulties with feeding and required placement of a gastrotomy tube in the first 2 years of life. Additionally, her facial anomalies had a significant impact on her ability to breathe normally and contributed to her OSA, documented on polysomnogram following adenotonsillectomy at age 5. The child required Bilevel Positive Airway Pressure (BiPAP) after surgery which resulted in significant improvement in her OSA**.** The child has also had chronic otitis media and chronic eustachian tube dysfunction, resulting in the insertion of multiple sets of tympanostomy tubes. Currently, the patient has a right marginal tympanic membrane perforation and recurrent episodes of otorrhea, especially from the right ear. Cultures of the otorrhea fluid have been positive for *Staphylococcus sp., Cornybacterium,* and *Alcaligenes*, but it is unclear if these were true pathogens or represented colonization of the ear canal. The most recent behavioral audiometric data, obtained in 2019, was not reliable, but distortion product otoacoustic emissions were present from 1500 to 6000 Hz in the left ear, consistent with normal function of the outer hair cells of the cochlea for these frequencies on the left; the right ear was not tested at that time due to otorrhea. An auditory brainstem response test was recommended due to the low reliability of the behavioral audiogram and inability to test the right ear, but has not been completed. The plastic surgeons are considering a midface advancement to improve her bite and improve nasal breathing, but there is concern that she will not tolerate this procedure.

## Discussion and conclusions

We presented the 14-year history of a child with acroscyphodysplasia, emphasizing the challenges of early clinical and radiographic diagnosis, with particular focus on the management of the complications associated with cone-shaped epiphyses. We also report a novel finding of premature growth arrest and tethering of the posterior distal femoral epiphysis. The existing literature emphasizes the genetics and pathophysiology of this disorder, but fails to address the significant impact that growth arrest, cone-shaped epiphyses, and the resultant deformities can have on patient mobility. It is also important to consider the ramifications of facial hypoplasia on the airway which in our patient contributed significantly to her severe OSA, ultimately requiring BiPAP.

There was a significant delay in making the correct genetic diagnosis. The possibility of chondrodysplasia punctata was considered clinically but rejected in the absence of epiphyseal stippling, as well as negative plasmalogen testing, thus ruling out peroxisomal defects as an underlying cause. Microarray analysis at age 2 raised the possibility of NF1 but she does not fulfill the diagnostic criteria: she has fewer than 6 café-au-lait lesions, does not have axillary or inguinal freckling, optic glioma, or evidence of any neurofibromas, plexiform neurofibromas, or Lisch nodules. Although she does have facial dysmorphism, it is due to a shallow facial profile, not sphenoid dysplasia. Similarly, there is no thinning of her cortical long bones or evidence of a pseudarthrosis. While she does have significant freckling and possible café-au lait spots, she does not meet the criteria for NF1. These finding can also be seen in patients with acrodysostosis or even AHO and PHP patients [13]. All three disorders can be associated with hyperpigmented lesions because they all have mutations that affect the same signaling cAMP pathway that is important in melanocyte differentiation [14]. Moreover, the NF1 microdeletion syndrome is typically characterized by a more severe clinical presentation than that seen in the majority of NF1 patients, with more cutaneous, subcutaneous and externally visible plexiform neurofibromas than age-matched patients with intragenic NF1 mutations, in addition to facial dysmorphic features, cognitive impairment, developmental delay, and an increased risk of malignant tumors [15–17]. This patient demonstrates developmental delay, is non-verbal but able to follow simple commands and has short stature, all of which can be found in NF1, but these features fit a wide variety of genetic disorders besides NF1 and acroscyphodysplasia. The skeletal, cranial, and endocrinologic features of this case are not consistent with NF1, but do support the diagnosis of acroscyphodysplasia.

Acrodysostosis was initially described in 1968 based on radiographic findings, but it was only in 2011 that variants in *PRKAR1A* were identified as its cause [18, 19]. Subsequently, additional variants in *PDE4D* were identified, thus expanding the genetic understanding of the disorder [5, 19, 20]. In addition, metaphyseal acroscyphodysplasia is known to be caused by PHP with associated *GNAS* mutations, or as a variation of acrodysostosis with *PDE4D* or *PRKAR1A* variants.

The skeletal findings in our patient at 7 months of age, although mild, included brachycephaly, mild coning of the right knee epiphysis, brachydactyly, and advanced bone age. A follow-up survey at 33 months of age showed more striking features, with progressive midface hypoplasia, brachycephaly, asymmetric epiphyses of the right knee and right proximal humerus, brachydactyly, as well as advanced bone age. The asymmetry of the cone-shaped epiphyses was confusing, suggesting a more focal physeal injury.

As previously reported, PHP is commonly found in patients with acrodysostosis and seems to play a major role in the premature closure of the epiphysis in some cases [1]. More specifically, the PTH receptor acts on chondrocytes and inhibits terminal differentiation, promoting physeal growth [8, 12]. Therefore, disruptions in the PTH signaling pathway, either in tissue unresponsiveness or in PTH production, may result in distinct cone-shaped epiphysis in the central portion of the growth plate [1]. It is important to note that our patient had relatively mild PHP, which was easily corrected with minimal doses of calcitriol and calcium carbonate. Thus, it is unclear how much of an impact the iPTH levels played in her advanced bone age, development of cone-shaped epiphyses or premature closure of the distal femoral posterior growth plates. An additional factor contributing to our patient’s skeletal advanced age, like many other patients with acrodysostosis, was a BMI in the 120th percentile. Estrogen, an important driver of skeletal maturity, can undergo aromatization in adipose tissue and may have contributed to the premature epiphyseal closure. On the other hand, although the patients is Tanner stage 5, she was still premenarchal at 13 years 8 months. Therefore, in the final analysis, it is unclear whether her endocrinological abnormalities played a role in the development of her skeletal deformities.

Although the term cone-shaped epiphyses appears frequently in the literature, we were unable to identify any reports summarizing the long-term sequalae of this physeal variant on limb growth. Cone-shaped epiphyses are often noted as a normal variant without long-term issues. They are described in numerous dysplasias/syndromes and can also be secondary to injury to the physis, either infection or infarction. In these cases, bone growth can be impacted, with resultant shortening and bowing, and images in the literature typically demonstrate central growth plate arrest leading to limb shortening. As noted earlier, we believe that the significant bowing of the right femur, and later on the left one, was due to closure of the growth plate posteriorly, leading to the need for extension osteotomies twice. Upon review, we question whether an effort at growth plate release might have been helpful; however, we would have been concerned about pathologic fracture since this child has severe intellectual disability and resists efforts to constrain her activity. Additionally, we were puzzled by the fact that posterior tethering of the growth plate occurred much later on the left leg. We hope that, in the future, a multi-site review of the cone-shaped epiphyses will shed further light on optimal care for this challenging problem.

In summary, this is a complex case of acroscyphodysplasia that demonstrates the challenges of genetic identification, as well as endocrine, otolaryngology, and orthopaedic management. Many of the phenotypic findings of acrodysostosis overlap with AHO and PHP, making a correct diagnosis difficult. Brachydactyly and cone-shaped epiphyses are classic radiographic findings that have been extensively described in the literature, and ideally a diagnosis can be made upon these features alone. However, now that the *PDE4D* and *PRKAR1A* genes have been identified as a cause of acrodysostosis, the diagnosis process has been simplified. For the orthopaedic surgeon, recognition of the natural history and deformities caused by cone-shaped epiphyses and premature growth arrest is essential in order to maintain normal limb alignment and protect mobility. Nonetheless, a multidisciplinary approach involving radiology, genetics, otolaryngology, orthopaedics, and endocrinology is crucial for the correct diagnosis and long-term management of these patients.

## Supplementary information

**Additional file 1.**

## Data Availability

The datasets generated during the current study are available as Supplementary Data Table 1.

## Abbreviations

AHO: Albright’s Hereditary Osteodystrophy
PHP: Pseudohypoparathyroidism type 1
PPHP: Pseudopseudohypoparathyroidism
OSA: Obstructive sleep apnea
PTH: Parathyroid hormone
*FGFR3*: Fibroblast growth factor
*ARSE*: Arylsulfatase E
*NF1*: Neurofibromatosis 1
LCL: Lateral collateral ligament
PTH: Parathyroid hormone
TSH: Thyroid-stimulating hormone
iPTH: intact parathyroid hormone
BiPAP: Bilevel positive airway pressure
